# Local population and regional environmental drivers of cholera in Bangladesh

**DOI:** 10.1186/1476-069X-9-2

**Published:** 2010-01-14

**Authors:** Michael Emch, Mohammad Yunus, Veronica Escamilla, Caryl Feldacker, Mohammad Ali

**Affiliations:** 1 Department of Geography, University of North Carolina-Chapel Hill, USA; 2 Carolina Population Center, University of North Carolina-Chapel Hill, USA; 3 ICDDR, B: Centre for Health and Population Research, Bangladesh; 4 International Vaccine Institute, Korea

## Abstract

**Background:**

Regional environmental factors have been shown to be related to cholera. Previous work in Bangladesh found that temporal patterns of cholera are positively related to satellite-derived environmental variables including ocean chlorophyll concentration (OCC).

**Methods:**

This paper investigates whether local socio-economic status (SES) modifies the effect of regional environmental forces. The study area is Matlab, Bangladesh, an area of approximately 200,000 people with an active health and demographic surveillance system. Study data include (1) spatially-referenced demographic and socio-economic characteristics of the population; (2) satellite-derived variables for sea surface temperature (SST), sea surface height (SSH), and OCC; and (3) laboratory confirmed cholera case data for the entire population. Relationships between cholera, the environmental variables, and SES are measured using generalized estimating equations with a logit link function. Additionally two separate seasonal models are built because there are two annual cholera epidemics, one pre-monsoon, and one post-monsoon.

**Results:**

SES has a significant impact on cholera occurrence: the higher the SES score, the lower the occurrence of cholera. There is a significant negative association between cholera incidence and SSH during the pre-monsoon period but not for the post-monsoon period. OCC is positively associated with cholera during the pre-monsoon period but not for the post-monsoon period. SST is not related to cholera incidence.

**Conclusions:**

Overall, it appears cholera is influenced by regional environmental variables during the pre-monsoon period and by local-level variables (e.g., water and sanitation) during the post-monsoon period. In both pre- and post-monsoon seasons, SES significantly influences these patterns, likely because it is a proxy for poor water quality and sanitation in poorer households.

## Background

Cholera is associated with regional environmental forces such as rainfall patterns, sea surface temperature (SST), and El Nino Southern Oscillation [1-6]. The spatial variation of cholera also varies substantially at a local level and this variation has been shown to be related to local environmental and population-level variables [7-9]. The objective of this study is to examine the role of regional environmental drivers of cholera while simultaneously considering the local level variation of socio-economic status (SES). SES is a proxy for more proximate variables associated with cholera etiology such as availability of clean water and the sanitation environment. Regional environmental variables considered include (SST), sea surface height (SSH), and ocean chlorophyll concentration (OCC), all of which have been shown to be related to cholera fluctuations [3,10,11]. The study is conducted in rural Bangladesh where we collect laboratory confirmed cholera cases and detailed SES data at the household level. These data are integrated with times series of SST, SSH, and OCC that we compile from satellite sensor data. We investigate whether SES modifies the effect of regional environmental forces on cholera incidence in Matlab.

## Materials and methods

**Figure 1:**
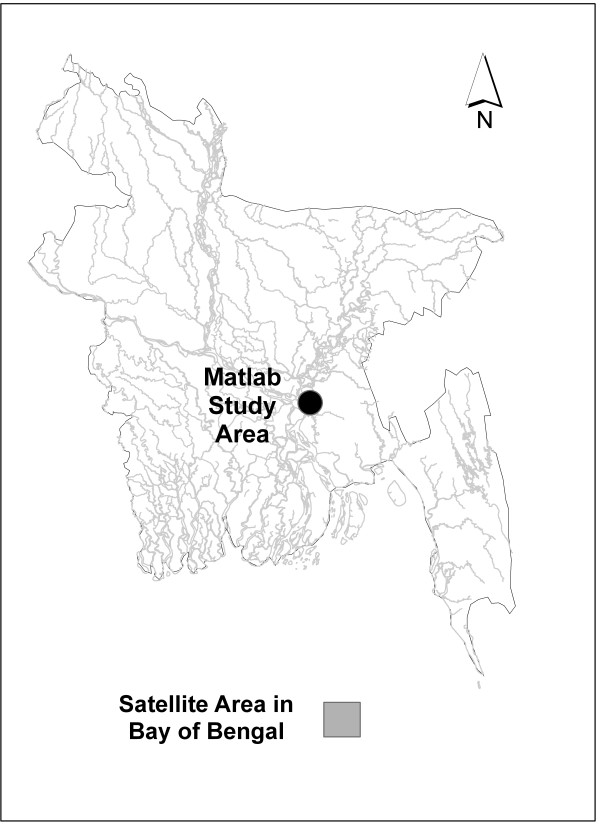
**Bangaldesh study area**.

**Figure 2:**
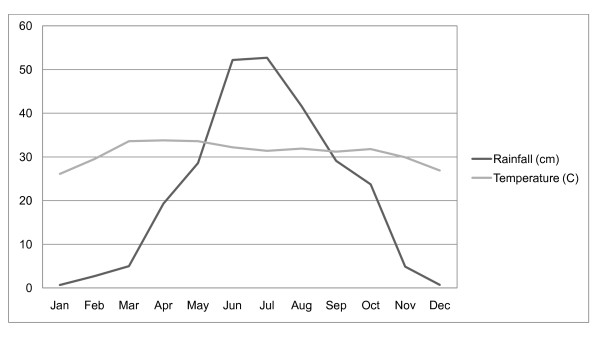
**Bangladesh monthly temperature and rainfall**.

The study is conducted at the field research site of the International Centre for Diarrhoeal Disease Research, Bangladesh (ICDDR, B) in Matlab, Bangladesh. Matlab is located in south-central Bangladesh approximately 50 km southeast of Dhaka near the Lower Meghna River (Figure 1). The study area has a typical monsoonal climate. Figure 2 shows the seasonal rainfall and temperature patterns in the study area. The temperature is coolest in January with an average high of 26 degrees Celsius and it gets hotter gradually each month until June when the monsoon begins. During January, rainfall levels are lowest with less than a centimeter on average. Rainfall gradually increases until approximately August, decreasing gradually thereafter each month until the end of the year.

The study-area population is approximately 200,000 for which a health and demographic surveillance system (HDSS) has been in place since 1966. A community health worker regularly visits each household in the study area and collects information about demographic events. Patients with severe diarrhea are referred to the ICDDR, B hospital. Each patient admitted to the Matlab hospital has a stool sample taken and pathogens are identified in a laboratory. A detailed census conducted in 2005 collected additional information about household SES including all assets. A GIS database was created at the household-level (i.e., partilineally-related extended household units called baris), allowing us to link disease incidence and SES information with specific household locations.

Table 1 summarizes all study variables and data sources. The satellite data for SST is distributed by NASA's Jet Propulsion Laboratory. The Advanced Very High Resolution Radiometer (AVHRR) sensor collects data for 4-km areas. This analysis is based on 8 × 8 pixel areas: each record represents an average value for a 32 km × 32 km (1024 km^2^) area. SSH, a measure of sea-level anomalies is derived through satellite altimetry from the Jason-1 sensor. OCC is available from SeaWiFS, a sensor that remotely measures chlorophyll concentration at a spatial resolution of 9 km. The satellite-derived, monthly environmental variables for all 3 sensors are compiled from the same geographic area in the Bay of Bengal (Figure 1). Data are available for all study variables from January 2003 to December 2007, the time period of analysis.

**Table 1:** Study variables and data sources

Variable	Data Source
Ocean chlorophyll concentration	SeaWiFS

Sea surface temperature	AVHRR

Sea surface height	Jason-1

Socio-economic status	Matlab census

Cholera case data	ICDDR, B Hospital

The analysis is based on monthly cholera cases for 6,840 baris for a five-year study period (2003-2007), a total of 410,400 observations. Although the cholera time series is available before that time period, study data are limited to this 5-year period because data for the Jason-1 sensor are available only from January 2003. A categorical SES variable was developed using principle components analysis (PCA) in STATA 10.0, creating a single household-level SES measure from multiple census variables. The SES measure reflects a composite of five dummy variables of ownership of household assets and one ordinal variable of household wall material (Table 2). Roof material was also collected but was excluded because most residents have a tin roof. Household-level SES scores were then collapsed by bari, and the mean score represents bari-level SES. All bari-level SES scores were sorted from lowest to highest and divided into equal quintiles. Higher quintiles reflect higher SES. It is hypothesized that households with lower SES have a greater risk of cholera transmission during seasonal outbreaks.

**Table 2:** Variables included in the PCA to calculate the SES score

Household Assets	Wall Material
Quilt/blanket	1 = Pucca (Mixed)

Hurricane lamp	2 = Tin Wall

Watch	3 = Tin (Mixed)

Bed	4 = Wood (Mixed)

Bicycle	5 = Bamboo (Mixed)

	6 = Other

	7 = No Dwelling

Relationships between cholera, environmental variables, and SES are measured using generalized estimating equations (GEE) with a logit link function to account for the bari-level correlation over time [12,13]. The models are built using independent and exchangeable within-bari correlation matrices to control for the correlation. The dependent variable is occurrence of cholera (yes or no) during each analyzed bari-month. The environmental and SES variables are explanatory variables. Coefficients of independent variables in the models are exponentiated to estimate the odds ratio (OR) of cholera associated with different variables. Standard errors for coefficients are used to estimate P values and associated 95% confidence intervals (95% CI) for the ORs. All statistical tests are interpreted using a two-tailed distribution. Multivariate models include the variables shown in Table 1 as well as variables for season and year. Two separate models are built to differentiate between the two main cholera epidemics in Matlab, one before and one after the monsoon [8].

## Results

**Figure 3:**
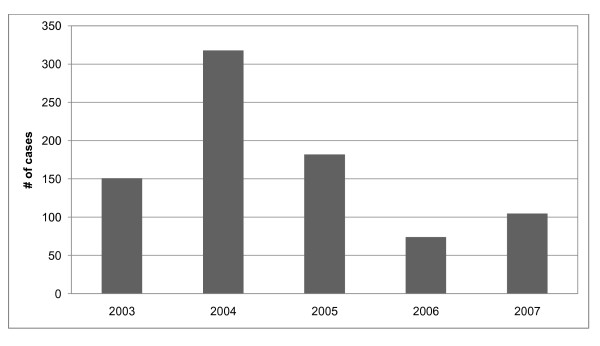
**Annual distribution of cholera cases in Matlab, Bangladesh: 2003-2007**.

**Figure 4:**
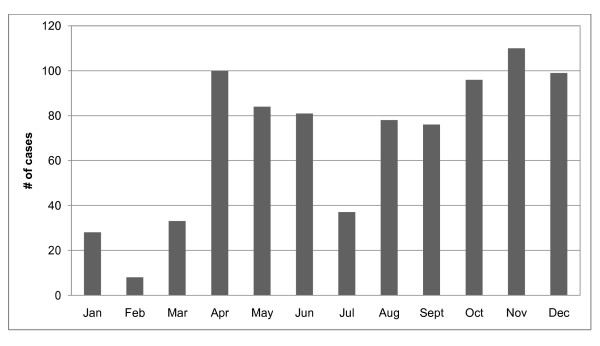
**Monthly distribution of cholera cases in Matlab, Bangladesh: 2003-2007**.

**Figure 5:**
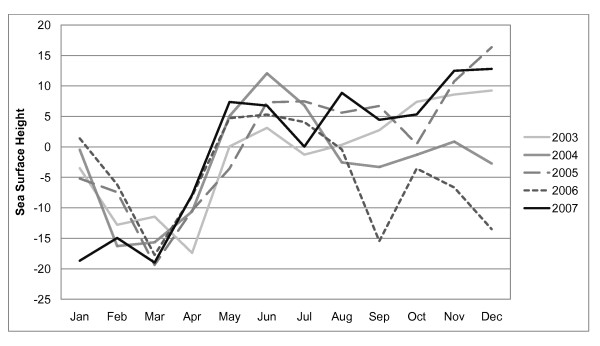
**Monthly SSH for each of the five study years**.

**Figure 6:**
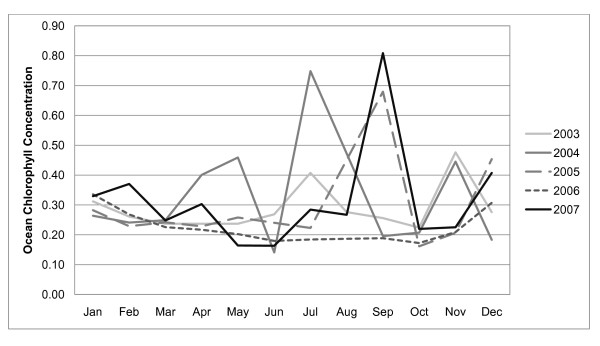
**Monthly OCC for each of the five study years**.

**Figure 7:**
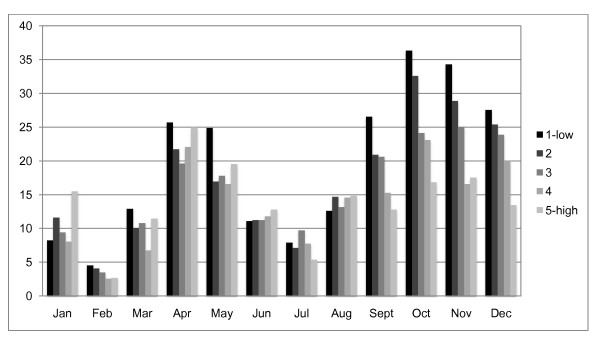
**Cholera cases per 100,000 in Matlab, Bangladesh by SES, per month: 1983-2007**.

From January 2003 to December 2007, there were 830 laboratory-confirmed hospitalized cholera cases in the study area. Figure 3 shows the distribution of cases by year. Monthly fluctuations of cholera cases since 2003 are displayed in Figure 4. Increases in reported cases are evident during the pre- and post-monsoon months. Figure 5 shows the variation in SSH by month for each of the five years. Figure 6 shows the same for OCC. Cholera cases stratified by SES score for the past 24 years (1983-2007) are presented in Figure 7. There is no apparent pattern during most of the year. However, during the post-monsoon epidemic period, there are many more cholera cases in lower socioeconomic households.

The lowest occurrence of the disease is in the winter (January to March) (Figure 4). Cholera was four times higher during the summer (April-June) and autumn (October-December), which is before and after the monsoon season respectively. During the monsoon season (July-September), the occurrence of cholera was two times higher than in the winter season.

Table 3 shows the results of the multivariate model estimating the risk of cholera by bari. SES is significantly associated with the occurrence of cholera: the higher the SES score, the lower the occurrence of cholera. SSH is significant, and negatively associated with cholera incidence. OCC is positively associated with the incidence of cholera in Matlab. SST is not related to cholera incidence.

**Table 3:** Predictors of cholera risk in baris in Matlab, Bangladesh

Variables	OR*	95% CI	p-value
Year	0.77	0.73 - 0.80	< .001
Season 2 (Apr-Jun)^†^	4.05	2.55 - 6.42	< .001
Season 3 (Jul-Sep)^†^	2.16	1.39 - 3.34	< .001
Season 4 (Oct-Dec)^†^	4.56	3.08 - 6.76	< .001
Socioeconomic score	0.77	0.72 - 0.84	< .001
SST	0.99	0.91 - 1.07	.867
SSH	0.98	0.97 - 0.99	.006
OCC	2.42	1.13 - 5.17	.023

* Multivariate odds ratio for the cited variable, adjusted for all other variables in the table, in a model using Generalized Estimating Equations (GEE) with the logit link function.

^† ^Reference category is season 1 (Jan-Mar).

Table 4 shows the results of separate models built for the pre- and post-monsoon periods. In both models, SES has a significant impact on the occurrence of cholera: in higher SES baris, there is less cholera. There is also a significantly negative association between cholera and SSH during the pre-monsoon period; however, this relationship does not hold for the post-monsoon period. OCC is positively associated with cholera during the pre-monsoon period but not for the post-monsoon period. SST is not related to cholera incidence during either period.

**Table 4:** Separate Models for pre- and post-monsoon epidemics

Variables	OR	95% CI	P-value
Pre-monsoon (April-June)			

Year	0.84	0.75-0.93	0.0010**

SES	0.81	0.70-0.94	0.0053**

SST	0.96	0.72-1.27	0.7827

SSH	0.98	0.96-0.99	0.0215*

OCC	1.82	1.19-2.79	0.0054**

Post-monsoon (October to December)			

Year	0.78	0.73-0.83	< .0001**

SES	0.78	0.70-0.88	< .0001**

SST	0.91	0.84-1.00	0.0555

SSH	0.98	0.97-1.00	0.1178

OCC	0.48	0.15-1.49	0.2066

** Significant at 0.01 level *at 0.05 level

## Discussion and Conclusions

Cholera is related to the regional environment but the relationship is stronger during the pre-monsoon period. In rural Bangladesh cholera is rare during the winter when the temperature is low and there is little rainfall. The temperature gradually gets hotter and rainfall remains low until the monsoon arrives. At the end of the hot, dry period, there is a pre-monsoon epidemic almost every year. This study shows that OCC and SSH are related to cholera at the bari-level during this pre-monsoon period and poorer people are more likely to contract the disease. During the actual monsoon period, there is less cholera, which has been attributed to a dilution effect that reduces the amount of bacteria in the aquatic environment [10]. Following the monsoon, the epidemic is much larger than the pre-monsoon epidemic. During the post-monsoon period, the regional environmental variables are no longer important. SES remains important throughout the year. The results of our analysis illustrate a very clear pattern of disparities between richer and poorer people during this post-monsoon period. This is likely because poorer people have less access to clean water and improved sanitation.

Cholera transmission is divided into primary and secondary types [7,14]. Primary cases are the result of infection by surface water sources, such as when people are directly infected with the bacteria that cause cholera by drinking untreated water. Primary transmission is influenced by factors such as temperature, salinity, and nutrient concentrations, which are related to environmental parameters. Secondary cases consist of people who are infected through fecal-oral transmission, such as when a family member is infected by a sick member of his/her family when the sick person puts his/her contaminated hands in the family's drinking water pot. Secondary transmission is related to poor water and sanitation environment, thus illuminating why there are socio-economic disparities with cholera.

SES is likely correlated with many variables responsible for cholera including the water and sanitation environment as well as educational level. Poorer people have fewer financial resources to invest in sanitary latrines with simple septic systems (i.e., cement ring latrines with septic holding tank) or cleaner water sources (i.e., deep tube wells). This is consistent with an earlier study by Emch [7] which found that cholera is more common in poorer households with less access to tubewell water and sanitary latrines. The finding that SES modifies the effect of the environment in a very regular pattern during the largest annual cholera epidemic (October through December) is quite remarkable given that most of the population in rural Bangladesh is very poor. Thus, what this study shows is that there are still disparities in cholera occurrence at the bottom end of the SES distribution. The policy implication of this finding is that local level poverty alleviation programs such as improving sanitation and drinking water access will likely have an impact on cholera, especially during the large annual epidemic in the post-monsoon season during which secondary cholera transmission is more common.

The main contribution of this project is that it describes how local population factors (i.e., SES) modify the effect of regional environmental forces on cholera. These findings must be considered in the context of previous research that describes cholera fluctuations in relation to climate and environment. In contrast to Lobitz et al. [3], the present study did not find a statistically significant relationship between cholera and SST in multivariate models. The main differences between that study and our's is that we included SES with the satellite-derived environmental variables in multivariate models and the studies were conducted during different time periods. The Lobitz et al. [3] study investigated cholera from 1992 to 1995 which was one of the highest cholera incidence periods in Matlab during the past 25 years. Lobitz et al. [3] found a positive relationship between SSH and our study found no relationship during the post-monsoon season and a weak relationship during the pre-monsoon season. It is unclear why the relationship is different and the underlying mechanism is not well understood. Constantin de Magnya et al. [11] found that cholera was positively related to OCC in both Kolkata, India and Matlab, Bangladesh, which is consistent with our study. We however built separate models for different seasons and found that the relationship exists between OCC and cholera only during the pre-monsoon period. Pascual et al. [2] investigate SST using the Niño 3.4 index which measures anomalies in a region of the equatorial Pacific. Thus, it investigates cholera and SST at a very different spatial scale than the present study.

This paper leads us to suggest that cholera is driven by regional environmental drivers during the pre-monsoon period and controlled more by local socio-economic drivers (e.g., water and sanitation) during the post-monsoon period. It is also notable that everyone in this study area is very poor by industrialized country standards. Therefore, it follows that in a larger distribution of SES that includes much richer households, these patterns of cholera disparities would likely be even stronger. At the same time, the environmental patterns during the pre-monsoon period would only be relevant for quite poor people, such as those living in the Matlab area, as richer people are able to better buffer themselves from the environment and, therefore, from contracting environmental diseases like cholera.

## Abbreviations

OCC: Ocean Chlorophyll Concentration; SES: Socio-economic Status; SSH: Sea Surface Height; SST: Sea Surface Temperature; ICDDR, B: International Centre for Diarrhoeal Disease Research, Bangladesh; HDSS: Health and Demographic Surveillance System; AVHRR: The Advanced Very High Resolution Radiometer; PCA: Principle Components Analysis; GEE: Generalized Estimating Equations.

## Competing interests

The authors declare that they have no competing interests.

## Authors' contributions

ME conceived of and designed the study, analyzed the data, and drafted the manuscript. MA participated in the study design, database management, and analysis. MY participated in the study design and data collection. VE participated in the database management and variable construction. CF participated in the database management and variable construction. All authors read and approved the final manuscript.
